# Enhanced patient counselling and SMS reminder messages to improve access to community-based eye care services in Meru, Kenya: statistical analysis plan for a Bayesian adaptive trial

**DOI:** 10.12688/wellcomeopenres.23495.1

**Published:** 2025-02-13

**Authors:** Min Jung Kim, Luke Allen, Malebogo Tlhajoane, David Prieto-Merino, Nigel Bolster, Andrew Bastawrous, David Macleod

**Affiliations:** 1 Faculty of Epidemiology and Population Health, London School of Hygiene & Tropical Medicine, London, UK; 2 Faculty of Infectious and Tropical Diseases, London School of Hygiene & Tropical Medicine, London, UK; 3 Universidad de Alcala, Madrid, Spain; 4 Peek Vision, London, UK; 5 International Centre for Eye Health, London School of Hygiene & Tropical Medicine, London, UK

**Keywords:** Adaptive trial, Bayesian trial, interim analysis, stopping rules, health services research, statistical analysis plan

## Abstract

**Background:**

Health service programmes frequently encounter challenges with patient adherence to care. A promising, low-risk approach to address this issue is providing patients with targeted information about the importance of adherence. In the Vision Impact Project (VIP), an eye health screening programme in Kenya, the adherence rate to attending triage clinics after referral is around 50%. To improve this rate, this trial will test the effectiveness of delivering relevant information to patients at the point of referral along with reminder messages.

**Methods:**

A pragmatic, Bayesian adaptive trial will be conducted within the VIP programme to assess the effectiveness of providing enhanced information compared to standard care. Weekly interim analyses will monitor adherence rates to referral appointments following positive vision impairment screenings. Participants will be randomized equally into intervention and control groups. The trial will stop if interim findings indicate either efficacy of one arm over the other, or futility that the two arms are performing equally.

**Discussion:**

This paper presents the statistical analysis plan for a pragmatic adaptive trial aimed at improving adherence in an eye screening programme in Kenya. This statistical analysis plan expands on the design and analysis plan detailed in the study protocol and documents decision rules to avoid post hoc decision-making.

**Trial registration:**

ISRCTN 11329596, Registered on 02 February 2024, https://doi.org/10.1186/ISRCTN11329596

## Plain language summary

This study is taking place in Meru, Kenya, as part of the Vision Impact Project (VIP). VIP is an eye health screening programme that identifies people living with vision impairment and refers them for appropriate care. One major challenge with the programme is that only about half of those people identified with vision impairment attend their referral appointments. This low rate of follow-up care is a common issue observed in health programmes globally.

To address this, we are conducting a study to test if providing extra information and sending reminder SMS messages can encourage more patients to attend their referral appointments. Patients will be randomly assigned to one of the two groups. Half will receive the additional information and SMS reminders, while the other half will receive the usual care that excludes these extras. Each week we will compare the attendance rates between the two groups to see if the added information and reminders improve attendance. If we find strong evidence that either one of the groups is performing better, or that both groups are performing equally, we will stop the study early and draw a final conclusion.

This study uses a more flexible and responsive approach than traditional trial designs. This study is designed to quickly and accurately identify which group is doing better when there is a real difference, even if the difference is small. While this method carries a relatively high chance of mistakenly concluding that one group is better when there is actually no difference, we have accepted this trade-off. We believe the risks of this higher chance of false conclusions are minimal because both patient groups are receiving low-risk, low-cost care. This study approach could also be useful for other health programmes trying to find effective ways to improve patient adherence.

## Introduction

### Background and rationale

Health service programmes often face challenges in improving patient adherence, particularly in screening initiatives designed to identify and connect patients with care. This issue is evident in vision impairment (VI) screening, where timely access to care is crucial for enhancing quality of life and preventing avoidable complications such as blindness. Peek Vision ( https://peekvision.org), a social enterprise, aims to address the global burden of VI by providing eye screening and patient management tools that facilitate referrals to local healthcare systems. To date, Peek Vision has screened over eight million people worldwide, identifying about 1.6 million in need of eye care. Despite the efforts, internal data shows that adherence rates are around 50%, revealing a gap in connecting patients to care.

In the Vision Impairment Project (VIP) based in Kenya, which employs Peek Vision’s screening platform, similar trends have been observed. Young adults aged 18–44 show particularly low adherence rates around 30%, highlighting the need for targeted interventions for this group. Interviews and surveys with these young adults have identified key barriers and potential solutions to improve adherence ^1^. One promising strategy is to provide information about the importance of care to increase awareness and encourage health-seeking behaviour.

These service modifications are low-risk and expected to yield modest improvements on attendance. Traditional, fixed-duration trials to test such incremental changes could be resource-intensive and time-consuming. As an alternative, we will conduct a pragmatic adaptive trial within the VIP programme, leveraging accumulating data and early stopping rules. The trial will assess whether providing additional information at the point of referral and via SMS reminders can enhance adherence and increase the proportion of people attending triage clinics. The trial setting, eligibility criteria, intervention definitions, and outcomes measurements have been described in detail in the study protocol ^2^. This paper expands on the protocol by providing a detailed statistical analysis plan.

### Objectives

To evaluate whether providing information about the importance of care increases the attendance rates in patients compared to those receiving standard care

### Trial design

This study is a pragmatic, Bayesian adaptive two-arm parallel trial embedded within the VIP programme. This trial was registered with ISRCTN on 2 Feb 2024 (registration number: ISRCTN11329596; DOI: https://doi.org/10.1186/ISRCTN11329596).

## Trial objectives and design

The detailed methodology of the trial, including eligibility criteria, methods of enrolment, and provision of the intervention, has been described in the study protocol ^2^.

### Study setting

This is a pragmatic trial embedded within the VIP programme operating in Meru, Kenya. It integrates the eye screening tool developed by Peek Vision, with data being managed on its patient management software.

### Eligibility criteria

This study will enrol adults (>18 years) who access the VIP programme at their local clinics, have been screened positive for vision impairment, and have consented to participate in the trial.

### Interventions

Participants will be randomly assigned to one of two groups in equal numbers. Both groups will be read a script from a screener at the point of referral and receive reminder SMS messages. The control group will receive standard referral counselling and two standard reminder SMS messages, which will include the location and date of the appointment. The intervention group will receive enhanced referral counselling and three reminder SMS messages, including an extra reminder message on the day of the appointment. Both the scripts and SMS messages received by the intervention group will include additional information that emphasizes the importance of eye care and the benefits of attending the referral. This trial aims to determine whether providing relevant information – through counselling, enhanced reminder messages, and increased message frequency – improves attendance rates compared to standard care.

### Outcomes

The programme will enrol all eligible adult participants. But the primary outcome will focus on the proportion of attendance in adults aged 18–44 years, who were identified in the formative research as the group less likely to attend appointments. Successful outcome will be defined as attending the scheduled appointment or within 14 days of the appointment date. A secondary outcome will measure overall attendance rates within 14 days of the appointment among all enrolled adults.

## Statistical methods

This trial design was conceptualised with two main objectives: to determine which of the two programmatic options to pursue, and to achieve this without necessitating an excessively large sample size, even in the case of only marginal differences between the arms. To meet these objectives, we will employ an adaptive trial approach, enabling real-time assessment of accumulating data throughout the trial. By comparing the results of interim analyses against prespecified stopping rules, we will potentially end the trial early if sufficient evidence is accrued. As this is an adaptive trial, participants will continue to be enrolled until one of the stopping criteria is met. While a target sample size is not defined, the trial will proceed for a maximum duration of one year if neither stopping rule is triggered.

### Interim analysis

Once the trial begins, interim analyses will be conducted every 7 days, with an average of about 300 adults aged 18–44 enrolled each cycle. The first interim analysis will take place after allowing a 14-day window for participants enrolled during the initial 7 days of the trial to attend their appointment on the scheduled date or within 14 days thereafter.

Bayesian methods will be used to analyse attendance in each arm. At each interim analysis, the proportion of attendees in each arm will be described using a binomial distribution of the outcome probability. This data will be combined with prespecified prior distributions through 10,000 Monte Carlo simulations to generate posterior distributions of the outcome proportions and the effect difference between the arms. These posterior distributions will be compared to predefined stopping rules to determine whether the trial should continue or stop. The decision to stop the trial will be solely based on the primary outcome, focusing on young adults aged 18–44.

We will use a uniform prior for the outcome probability of the control arm, specified as *logit(p) ~ norm(0, 0.3)*, reflecting variability between 0 and 1 ^3^. We will also use a neutral prior for the effect difference between the two arms, with an odds ratio of 1.0 and a 95% credible interval (95% CI) ranging from 1/30 to 30. This neutral prior allows for detection of a wide range of effect differences and ensures that the posterior distribution is primarily driven by trial data rather than specified prior beliefs ^4^, enhancing the generalizability of the results across various settings.

### Stopping rules

**Figure 1.**
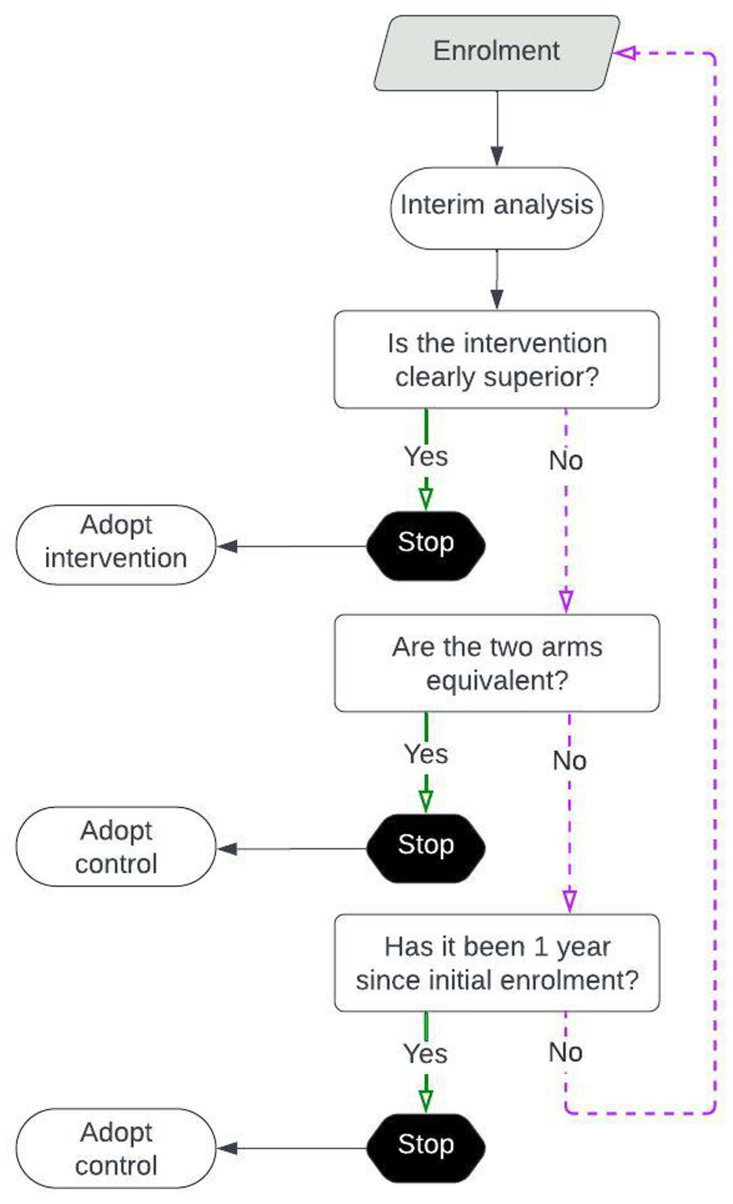
Decision-making processes of the trial. Interim analyses will be conducted every 7 days. At each analysis, all accumulated data will be evaluated according to two stopping rules: ( **a**) the stopping rule for efficacy, and ( **b**) the stopping rule for equivalence. If the intervention demonstrates clear superiority over the control, the trial will terminate and adopt the intervention arm. Conversely, if the control arm shows clear superiority over the intervention, the trial will stop and adopt the control arm. If the two arms show equivalent performance, the trial will stop and adopt the control arm. If neither stopping rule is met within one year of trial initiation, the trial will conclude and adopt the control arm.

During the trial design phase, two sets of stopping rules were established to determine when to stop the trial based on either efficacy or futility criteria. First, the trial will stop for efficacy if there is evidence that one arm is more effective than the other, indicated by an effect difference greater than 0%. The stopping rule for efficacy will be met if the posterior probability of an effect difference exceeds a threshold *E* (i.e. *P(effect difference* > 0%) ≥ *E*). When the trial stops for efficacy, the arm with the higher mean posterior distribution of the outcome proportion will be declared superior. Conversely, the trial will stop for futility if the two arms have equal or similar performance. The stopping rule for futility will be triggered if there is at least *F*% posterior probability that the effect difference between the two arms is smaller than *D* ( *P(effect difference* < *D* %) ≥ *F*), where *D* represents the maximum meaningful difference between the arms considered negligible, and F is the futility threshold for determining if sufficient evidence has been accrued ^5, 6^ ( Figure 1).

Simulations were conducted to determine the decision thresholds *E*, *F*, and *D* within optimal bounds, ensuring both adequate power and feasible sample sizes for the VIP programme. The simulations used datasets with small effect differences ranging from 0% to 5%, to configure thresholds for detecting small intervention effects. Various decision thresholds were tested, evaluating their impacts on performance measures including type I error, power, sample size, coverage, and bias.

Concurrently, the programme team discussed the level of evidence required to make decisions on the effectiveness of the intervention. This study is classified as a negligible risk trial with low-risk service modifications aimed at improving access to care. The team was willing to accept some chance of implementing the intervention if the two arms were equally effective. They also acknowledged a potentially small chance of adopting a marginally inferior arm, in order to prioritize a higher likelihood of adopting the intervention if it was indeed effective and to expedite the decision-making process within the trial.

Based on the simulation results and discussions, it was concluded that setting *E*=95%, *F*=95%, and *D*=1% provided sufficiently high power while maintaining manageable sample sizes for the VIP programme. Consequently, this trial will employ the following two stopping rules for the primary outcome:

(a) The trial will stop if there is evidence of efficacy, defined as any meaningful difference greater than 0% between the two arms. If the posterior probability of observing a difference meets or exceeds 95%, the arm with the higher mean posterior distribution of outcome proportion will be declared superior, prompting the trial to stop.
(b) Alternatively, the trial will stop if the difference between the two arms is negligible, defined as less than 1%. If there is at least 95% posterior probability that the difference is smaller than 1%, the trial will stop for futility, recognizing both arms as viable options.

Simulations using these stopping rules provided probabilities of adopting the intervention under different scenarios. The results indicate an 81.0% chance (power) of adopting the intervention if it increases attendance by 1%, with a median sample size of 3,800 (IQR [700-14,600]) required to achieve these results. If the intervention increases attendance by 3%, it will be correctly identified 94.3% of the time, with a median sample size of 1,300 (IQR [500-3,300]). In cases where there is no true difference between the two arms, the intervention would be adopted 36.3% of the time (type I error). If the intervention decreased attendance by 1% compared to the control arm, there is a 9.3% risk the intervention would be adopted.

### Final analysis

When one of the two stopping rules is met, the trial will terminate and proceed to the final analysis stage. The maximum duration of the trial is set at 1 year, with an estimated enrolment of 15,000 participants aged 18–44 years. If neither stopping rule is triggered within this timeframe, the trial will declare that the arms are equally effective, and the intervention will not be adopted. Upon completion of the trial, all accrued data will be analysed on an intention-to-treat basis.

***Baseline characteristics*.** Age and sex of the participants will be described according to allocation. Frequency and percentages will be reported for sex, while age will be summarized using mean and standard deviation. Data will be assessed for normality and checked for the presence of outliers.

***Primary and secondary outcomes*.** The primary outcome is attendance at the triage clinic *among young adults between ages of 18 and 44*, and the stopping rules are based only on the analysis of this group. The secondary outcome is the proportion of attendance in *all ages*. The final analysis will report the posterior probability of each arm being superior to another, and the posterior probability of the two arms having a negligible difference of 1% or less. Additionally, the posterior distributions of the outcome proportions in each arm will be described by mean and 95% CI. The posterior distribution of the effect difference between the arms will also be reported with mean and 95% CI ( Table 1).

**Table 1.** Reporting final analysis results.

	Probability of being superior	Probability of having a negligible difference	Mean success probability	Mean effect difference (95% CI)
18–44 years (primary outcome)				
Intervention arm				
Control arm				
44+ years				
Intervention arm				
Control arm				
All adults (secondary outcome)				
Intervention arm				
Control arm				

Posterior probabilities of being superior and having negligible difference of <1% will be reported for each age group. In each arm, the mean success probability and its 95% credible interval will be reported based on the posterior distribution of probabilities of success.

### Statistical software

All analyses will be conducted in R using the “rjags” package, which interfaces to JAGS library for Bayesian data analysis. Interim analysis will be automated within the Peek Vision’s data collection and analysis software, Peek Capture, which utilizes prewritten R scripts. Alerts will be issued by Peek Capture when a stopping rule is met. A statistician from the London School of Hygiene and Tropical Medicine will also perform weekly manual audits to verify the algorithm functions as intended.

## Discussion

This article presents the statistical analysis plan for our published trial protocol, which aims to evaluate whether providing information about the importance of care improves patient adherence. This statistical analysis plan was refined and finalized before the trial commenced.

This study is classified a negligible risk trial, focusing on evaluating the effect of low-risk service modifications within a health service programme. We have designed an adaptive trial to facilitate early decision-making, enabling prompt adoption of an intervention that may yield a marginal improvement in outcomes. Simulations have demonstrated that our study design is sufficiently powered to detect an intervention effect as small as 1% with a modest sample size, suitable for our programme setting. Nonetheless, our study design permits a type I error rate that is higher than typically acceptable in many clinical trials (36.3%) in scenarios where there is no intervention effect. This was deemed acceptable both by the programme and the ethics board due to the negligible risk of adverse events. We note that the magnitude of the relative effect observed should be interpreted with caution, as the early stopping rule for efficacy may potentially lead to overestimation of this measure.

Fixed-duration trials can be costly, especially when evaluating interventions with potentially small effects. To improve adherence, health service programmes often resort to before-and-after studies. Through this trial, we aim to demonstrate the value of this pragmatic adaptive trial design for evaluating low-risk service modifications, and to encourage research bodies to consider this approach for improve access and adherence in health service programmes.

## Study status

At the time of protocol submission, eligible participants have been recruited and had completed data collection.

## Ethics approval and consent to participate

The study received ethics approval from the Kenya Medical Research Institute (KEMRI) scientific and ethics review unit, and from the London School of Hygiene & Tropical Medicine research ethics committee on February 6, 2024 (reference no. 29549).

This was a pragmatic trial being implemented within an ongoing screening programme. The Institutional Review Board (IRB) approved the use of oral consent, considering the intervention to be low-risk and aiming to minimise disruption to the ongoing screening programme. Screeners obtained consent during the screening process and documented it electronically using a tick box.

## List of abbreviations

CI        Credible Interval

SMS    Short Message Service

VIP     Vision Impact Project

## Data Availability

No data are associated with this article. Open Science Framework: SPIRIT checklist for <Enhanced patient counselling and SMS reminder messages to improve access to community-based eye care services in Meru, Kenya: statistical analysis plan for a Bayesian adaptive trial> https://doi.org/10.17605/OSF.IO/T3YMF ^7^. Data are available under the terms of the Creative Commons Attribution 4.0 International license (CC-BY 4.0).
