# Supplementary material for: Enhanced patient counselling and SMS reminder messages to improve access to community-based eye care services in Meru, Kenya: statistical analysis plan for a Bayesian adaptive trial
Source: Wellcome Open Res. 2025 Feb 13;10:69. [Version 1] doi: 10.12688/wellcomeopenres.23495.1 (PMC11955077; doi:10.12688/wellcomeopenres.23495.1)
Supplement: Peer review history

# Reviewer report: Reviewer response for version 1

**DOI:** 10.21956/wellcomeopenres.25915.r118773

**Published:** 2025-03-29

**Authors:** Khin Than Win

**Affiliations:** 1 University of Wollongong, Wollongong, Australia

The study participants included those who had been screened positive for vision impairment. There can be various causes of vision impairment, and the urgency for referral may also differ. Are there any specific criteria to differentiate between positive and negative screening, or are there other categories? Based on that, the routine care would also be different.

 Please provide more information on the difference between the control and intervention. The objective of this study is to evaluate whether providing information about the importance of care increases attendance rates. 

 While the control group will receive standard referral counselling and two standard reminder SMS messages, the intervention group will receive enhanced referral counselling and three reminder messages. Are there any other features , attributes, or data collected that can help explain why attendance is delayed?

Is the study design appropriate for the research question?

Yes

Is the rationale for, and objectives of, the study clearly described?

Yes

Are sufficient details of the methods provided to allow replication by others?

Yes

Are the datasets clearly presented in a useable and accessible format?

Partly

Reviewer Expertise:

Health informatics, digital health, evaluation research

I confirm that I have read this submission and believe that I have an appropriate level of expertise to confirm that it is of an acceptable scientific standard.

# Reviewer report: Reviewer response for version 1

**DOI:** 10.21956/wellcomeopenres.25915.r119238

**Published:** 2025-03-25

**Authors:** Cathy Egan, Abraham Olvera-Barrios

**Affiliations:** 1 Moorfields Eye Hospital, London, UK; 2 Medical Retina, Moorfields Eye Hospital NHS Foundation Trust (Ringgold ID: 4960), London, England, UK

This is a pragmatic trial design with a high likelihood of rapid, data-driven, effective and actionable outcomes. The trial using a Bayesian adaptive design is of relevance to the field of eye screening and patient adherence to care. Some aspects of the manuscript could be refined to enhance clarity.

 One key potential problem with a text-based reminder system is literacy as a barrier to understanding the text. It was not clear from the description how this would be mitigated when the young adult was not in the presence of the healthcare worker.

 A second and potentially related problem would be vision impairment as a barrier to reading or understanding the text reminder. What is the risk that a small local variation in the screened population or the delivery could lead to either early stopping or early implementation? i.e. what measures are in place to monitor in real time that the screened population is representative of the general population? 

**Specific recommendations:**

- A breakdown on the justification of thresholds for increase in attendance would be useful. It is not entirely clear if small differences (which may be significant on Bayesian models, but may result in non clinically meaningful differences) would lead to early stopping and adoption of the intervention. 
- Please add a definition of "positive screen of visual impairment". 
- Discuss the effect of visual impairment impact on the ability of the participants to read the text messages been considered in the methodology.
- Discuss the level of literacy in the screened population and, if relevant, the potential impact on the results. Consider a mitigation strategy if relevant.
- A discussion on the decision to choose a 14-day window from appointment date to define a successful outcome would be informative. Is this realistic in practice on the screened population? i.e. how likely are participants to attend within 14 days after missing an appointment? 
- How will the data be handled if a participant misses multiple consecutive appointments in periods longer than the pre-specified 14-day window? 
- Given frequentist approaches tend to be more likely used in this context, further details on the justification of the choice and strengths of the Bayesian approach would be really informative in the background section.
- Please add the corresponding reference for the `rjags` package.

Is the study design appropriate for the research question?

Yes

Is the rationale for, and objectives of, the study clearly described?

Yes

Are sufficient details of the methods provided to allow replication by others?

Yes

Are the datasets clearly presented in a useable and accessible format?

Yes

Reviewer Expertise:

Medical retina including diabetic retinopathy screening; AI for diabetic retinopathy screening

We confirm that we have read this submission and believe that we have an appropriate level of expertise to confirm that it is of an acceptable scientific standard.
